# Prevalence, Predictive Factors, and Outcomes of Respiratory Failure in Children With Pneumonia Admitted in a Developing Country

**DOI:** 10.3389/fped.2022.841628

**Published:** 2022-05-04

**Authors:** Shamsun Nahar Shaima, Tahmina Alam, Abu Sadat Mohammad Sayeem Bin Shahid, Lubaba Shahrin, Monira Sarmin, Farzana Afroze, Irin Parvin, Sharika Nuzhat, Yasmin Jahan, Gazi Md. Salahuddin Mamun, Haimanti Saha, Mst. Mahmuda Ackhter, Md. Zahidul Islam, K. M. Shahunja, Sufia Islam, Tahmeed Ahmed, Mohammod Jobayer Chisti

**Affiliations:** ^1^Nutrition and Clinical Services Division, International Centre for Diarrhoeal Disease Research, Bangladesh (icddr,b), Dhaka, Bangladesh; ^2^Institute for Social Science Research, The University of Queensland, Brisbane, QLD, Australia; ^3^Department of Pharmacy, East West University, Dhaka, Bangladesh; ^4^Office of the Executive Director, International Centre for Diarrhoeal Disease Research, Bangladesh (icddr,b), Dhaka, Bangladesh

**Keywords:** outcome, respiratory failure, children, pneumonia, Bangladesh

## Abstract

**Background:**

Pneumonia has been the leading infectious cause of morbidity and mortality in children under 5 years of age for the last several decades. Although most of these deaths occur due to respiratory failure, published data are limited regarding predicting factors and outcomes of respiratory failure in children hospitalized with pneumonia or severe pneumonia.

**Objective:**

This study aimed to explore the prevalence, predicting factors, and outcomes of respiratory failure in children under-five with pneumonia or severe pneumonia.

**Methods:**

In this retrospective chart analysis, we enrolled children under 5 years of age hospitalized with pneumonia or severe pneumonia in the Dhaka Hospital of International Centre for Diarrheal Disease Research, Bangladesh (icddr,b) between August 2013 and December 2017. Comparisons were made between children with respiratory failure (*n* = 212) and those without respiratory failure (*n* = 4,412). Respiratory failure was defined when the oxygen saturation/fraction of inspired oxygen (SpO_2_/FiO_2_) was <315.

**Results:**

A total of 4,625 children with pneumonia or severe pneumonia were admitted during this study period. Among them, 212 (4.6%) children developed respiratory failure and formed the case group. A total of 4,412 (95.3%) children did not develop respiratory failure and formed the comparison group. In logistic regression analysis, after adjusting with potential confounders, severe sepsis [adjusted odds ratio (aOR): 12.68, 95% CI: 8.74–18.40], convulsion (aOR: 4.52, 95% CI: 3.06–6.68), anemia (aOR: 1.76, 95% CI: 1.20–2.57), and severe underweight (aOR: 1.97, 95% CI: 1.34–2.89) were found to be independently associated with respiratory failure. As expected, children with respiratory failure more often had fatal outcome than without respiratory failure (74, 1%, *p* < 0.001).

**Conclusion:**

The results of our analyses revealed that prevalence of respiratory failure was 4.6% among under-five children hospitalized for pneumonia or severe pneumonia. Severe sepsis, convulsion, anemia, and severe underweight were the independent predictors for respiratory failure in such children and their case-fatality rate was significantly higher than those without respiratory failure. Early recognition of these predicting factors of respiratory failure may help clinicians imitating prompt treatment that may further help to reduce deaths in such children, especially in resource-limited settings.

## Introduction

Pneumonia has been one of the preeminent causes of morbidity and mortality in children under 5 years of age for several decades (1–3). More than 90% of these deaths occur in developing countries involving two-thirds during the infancy period (1, 4). Estimated pneumonia-related deaths dropped down from 1.75 million in 2000 to 0.80 million in 2018. Still, pneumonia is responsible for 16% of a predicted 5.2 million global deaths among children less than 5 years of age (5, 6).

Several studies revealed that death from pneumonia is attributable to hypoxemia (7, 8), potentially due to respiratory failure. Most of the children having hypoxemia develop respiratory failure mainly due to ventilation-perfusion mismatch, derived from uncorrected hypoxemia (9). Indeed, the role of respiratory failure in childhood pneumonia is less focused in developing countries as all the emphases went to hypoxemia for its dreadful impact on childhood pneumonia-related deaths (10). Thus, understanding the prevalence, predicting factors, and outcomes of respiratory failure among children treated in the hospital for pneumonia or severe pneumonia is also critically important. In a study conducted in Taiwan among H1N1 influenza-related hospitalized pneumonia patients in 2009, 22/96 (23%) patients were found to have respiratory failure, where 10 (45%) patients died and all of the non-respiratory failure patients survived (11). Studies that were conducted among infants and children in critical care units of North American hospitals revealed that a good number of these children developed respiratory failure requiring mechanical ventilation. Total intensive care unit (ICU) admission for 6 consecutive months were 6,403, where 1,096 (17.1%) developed respiratory failure requiring mechanical ventilation within 24 h of development of respiratory failure. Among them, 97 had infantile pneumonia experiencing 1.6% mortality (12). A systematic review and meta-analysis reported that the most common causes of respiratory failure demanding mechanical ventilation in children were pneumonia and severe bronchiolitis (13).

Like other hospitals in developing countries, children in the Dhaka Hospital of International Centre for Diarrheal Disease Research, Bangladesh (icddr,b) often presented with pneumonia or severe pneumonia with other comorbidities of diarrhea, dehydration, sepsis, convulsion, and malnutrition (14). However, we did not find any published data on the predictors and outcomes of respiratory failure in children admitted with pneumonia or severe pneumonia, especially in developing countries such as Bangladesh. Thus, we attempted to understand the prevalence of respiratory failure among under 5 hospitalized children with pneumonia or severe pneumonia. Our further aim was to analyze the predicting factors and outcomes in such children.

## Materials and Methods

### Ethical Statement

Deidentified data were analyzed in this chart analysis; therefore, informed consent was not taken from the parents or guardians of the study participants. We have taken a waiver of the Ethics Review Committee (ERC) to use the deidentified data, which the ERC of icddr,b agreed and approved on 12 January 2020.

### Study Site and Design

This study was conducted in the Dhaka Hospital of icddr,b, the largest diarrheal disease hospital globally. This hospital provides care to an average of 160,000 patients of diverse ages each year, free of cost, 24 h a day, and 7 days a week. Inhabitants of Dhaka, mostly with poor socioeconomic demographics, used to visit this hospital. Generally, patients with diarrheal illnesses are admitted in the facility with or without complications associated with pneumonia, malnutrition, sepsis, and electrolyte imbalances (15). Initially, attending nurses at hospital triage obtain the medical history and rapidly evaluate the patients. Based on triage assessment, patients are either referred for physician‘s consultation or admitted to an appropriate ward of the hospital (7). After admission, a thorough assessment of the patients is performed by the attending physicians. Necessary investigations, as well as the management, including drugs and other supportive therapies, are given to the patients. Per year, on average, 1,500 patients are used to be treated in the intensive care unit (ICU) of this hospital and more than 60% admissions are due to pneumonia or severe pneumonia. The ward has the provision of portable chest radiograph, non-invasive ventilation, in particular locally made bubble continuous positive airway pressure (bCPAP) and mechanical ventilation. As per our national recommendation, vaccines, including *Haemophilus influenzae* type B (Hib) vaccine and pneumococcal conjugate vaccine (PCV), are provided free of cost (16). In this retrospective chart analysis, we enrolled children under 5 years of age hospitalized with pneumonia or severe pneumonia in the in-patient wards [acute respiratory infection (ARI) ward] and ICU of the Dhaka Hospital of icddr,b between August 2013 and December 2017. Children with respiratory failure were constituted as cases (*n* = 212) and those without respiratory failure formed the comparison group (*n* = 4,412).

### Sample Size and Sampling Technique

As it was an exploratory study, we collected data on children with pneumonia and severe pneumonia those were admitted into ARI ward or ICU during the period between 2013 and 2017. The children were divided into the two groups, with (*n* = 212) and without (*n* = 4,412) respiratory failure.

### Study Population

In this study, children had clinical pneumonia or severe pneumonia (17) with radiological confirmation (18) on admission. In children with cough or breathing difficulty, also having age-specific fast breathing or lower chest indrawing (with no danger signs of severe pneumonia) are being considered to have clinical pneumonia (17). Age-specific fast breathing in a child is defined as, if respiratory rate of ≥60 breaths/min for <2 months, ≥50 breaths/min for 2–11 months, and ≥40 breaths/min for 1–5 years (17). Severe pneumonia is defined as, if children aged >2–59 months had a history of cough or breathing difficulty plus hypoxemia (oxygen saturation <90%), central cyanosis, grunting, or signs of pneumonia with a general danger signs, such as unable to drink or breastfeed, lethargy or reduced level of consciousness, or convulsions (17). According to the WHO, if auscultatory findings showed diminished or bronchial breath sounds or signs of consolidation or other infiltrates or pleural effusion, it is considered as radiological pneumonia (18). Respiratory failure was defined when a study child was evaluated to have severe respiratory difficulty and hypoxemia having oxygen saturation/fraction of inspired oxygen (SpO_2_/FiO_2_) less than 315. For calculating FiO_2_ that the patient was receiving oxygen, we added 4% for each liter of oxygen to the FiO_2_ in room air (21%) (19).

### Patient Management

All the children with pneumonia were given standard treatment, following hospital guidelines described elsewhere (7). Children who had severe respiratory distress received 4 hourly monitoring of clinical signs of respiratory distress, including SpO_2_. They all received the WHO standard management for pneumonia and severe pneumonia, including parental antibiotics and nasogastric feeding (7). Chisti et al. evaluated the efficacy of locally made bCPAP compared to the WHO standard low-flow oxygen therapy in treating childhood severe pneumonia with hypoxemia in under-five children and it revealed that bCPAP was related with significant reduction of death (7). Mechanical ventilation was considered for those who failed with bCPAP.

### Measurements

We extracted data using existing electronic medical record system of the Dhaka Hospital of icddr,b. We identified and documented pneumonia or severe pneumonia-related occurrence during hospitalization upon the data availability. We collected data regarding children’s demographic information, immunization, and nutritional status. Moreover, data including clinical features, such as diarrhea, dehydration (some/severe), fever, congenital heart disease, severe pneumonia, convulsion, and severe sepsis were also collected on admission. Nutritional status was evaluated by calculating z-score (weight for height/length) with or without pedal edema following the WHO guidelines for childhood malnutrition (20). We also collected laboratory data to diagnose hyponatremia (21), hypernatremia (22), hypokalemia (23), hyperkalemia (24), and metabolic acidosis (25).

### Data Analysis

For data analysis, we used SPSS for Windows (version 20.0; SPSS Incorporation, Chicago, IL, United States) and Epi Info (version 7.0, USD, Stone Mountain, GA, United States). Data were first extracted from hospital patients’ electronic database and then plotted into these software for data management and analysis. Prevalence was calculated based on the representation of respiratory failure against the proportion of total pneumonia cases. On the other hand, the chi-squared (χ^2^) test was performed to measure the potential differences of clinical attributes in children having pneumonia or severe pneumonia with and without respiratory failure. In case of continuous variables, the Student’s *t*-test was used to compare the means of normally distributed data. We have used the Mann–Whitney *U*-test for the non-normally distributed data expressed in median and interquartile range (IQR). “*p*”-value of less than 0.05 was considered as statistically significant. Strength of association was determined by calculating odds ratio (OR) and their 95% CIs. The univariate analyses were done to identify the association between dichotomous variables. The multivariate logistic regression was done to adjust the possible confounders and to see the associated factors with respiratory failure among this study children. In the regression model, both the dependent (respiratory failure) and independent variables (significantly associated factors) were shown separately. It is important to note that except outcomes, all the analyzed symptoms were present on admission.

## Results

**FIGURE 1:**
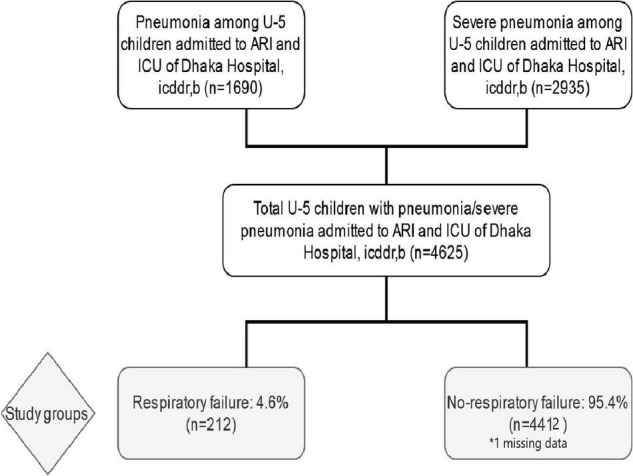
Study profile of the under-five children admitted with pneumonia or severe pneumonia with and without respiratory failure.

During this study period, a total of 4,625 children under 5 years of age with pneumonia or severe pneumonia were admitted to both the ARI and ICU. Among them, we found 36.54% (*n* = 1,690) had pneumonia, 63.46% (*n* = 2,935) had severe pneumonia, and 4.58% (*n* = 212) had respiratory failure. However, the prevalence of respiratory failure was 7.22% among children having severe pneumonia. A total of 4412 study children who did not have respiratory failure and, thus, constituted the comparison group (Figure 1).

Compared to study children without respiratory failure, the children with respiratory failure were more likely to have lack of immunization, having severe underweight, severe sepsis, convulsion, dehydration, anemia, and higher white blood cell count at admission (Table 1).

**TABLE 1:** Admission characteristics of under-five children who developed respiratory failure compared to those without respiratory failure hospitalized for pneumonia or severe pneumonia.

Parameters	Children with respiratory failure (*n* = 212)	Children without respiratory failure (*n* = 4412)	OR (95% CI)	*p*-value
Age in months (median, IQR)	7.56 (4.8, 11.2)	8.0 (4.9,12.1)	–	0.238
Duration of fever on admission (median, IQR)	3.0 (2.0, 5.0) (*n* = 131)	3.0 (2.0, 5.0) (*n* = 2269)	–	0.627
Duration of cough on admission (median, IQR)	4.0 (3.0, 7.0) (*n* = 84)	4.0 (3.0, 7.0) (*n* = 2775)	–	0.819
Immunized as per EPI schedule	126/170 (74.2)	2727/3320 (82.2)	0.62 (0.44–0.89)	0.009
Severe under-weight	121/211 (57.3)	1160/3890 (29.8)	3.16 (2.38–4.19)	< 0.001
Congenital heart disease	15 (7.1)	271/4410 (6.1)	1.16 (0.67–1.99)	0.583
Severe sepsis	132 (62.3)	268 (6.1)	25.51 (18.82–34.57)	< 0.001
Convulsion	93/211 (44.1)	421/3890 (10.8)	6.49 (4.85–8.67)	< 0.001
Ileus	9/211 (4.3)	207/3890 (5.3)	0.79 (0.40–1.56)	0.500
Diarrhea	188 (88.68)	3518 (79.74)	1.99 (1.29–3.06)	0.002
Dehydration	81/211 (38.4)	852/3890 (21.9)	2.22 (1.66–2.96)	< 0.001
Anemia	72/202 (35.6)	743/3266 (22.7)	1.88 (1.39–2.53)	< 0.001
Total WBC count	15990 (11010, 25410) (*n* = 201)	14310 (10280, 19250) (*n* = 3259)	–	< 0.001

*n, number of subjects; IQR, Inter-quartile range; EPI, expanded programme on immunization; OR, odds ratio; CI, confidence interval; WBC, white blood cell.*

During hospitalization, 156 (73.6%) children died with respiratory failure, in comparison 58 (1.3%) died among those who did not have respiratory failure and the difference was statistically significant (Table 2).

**TABLE 2:** Consequences of respiratory failure in children under 5 years of age compared to those without respiratory failure who were hospitalized for pneumonia or severe pneumonia.

Parameters	Children with respiratory failure (*n* = 212)	Children without respiratory failure (*n* = 4412)	OR (95% CI)	*p*-value
Duration of hospital stay (median, IQR)	4.0 (2.0, 7.0)	6.0 (4.0, 9.0)	–	<0.001
Death	156 (73.6%)	58 (1.3%)	209.12 (140.11312.10)	<0.001

*n, number of subjects; IQR, inter-quartile range; OR, odds ratio; CI, confidence interval.*

In the logistic regression analysis after adjusting for potential confounders, severe sepsis, convulsion, anemia, and severe underweight were found to be independently associated with respiratory failure (Table 3).

**TABLE 3:** Results of the multivariate logistic regression analysis to explore the independent predictors of respiratory failure in under-five children hospitalized with pneumonia or severe pneumonia.

Parameters	aOR	95% CI	*p*-value
Immunization as per EPI schedule	0.87	0.57–1.34	0.529
Severe underweight	1.97	1.34–2.89	< 0.001
Severe sepsis	12.68	8.74–18.40	< 0.001
Convulsion diarrhea	4.52	3.06–6.68	< 0.001
	0.64	0.35–1.16	0.144
Dehydration	1.32	0.89–1.94	0.160
Anemia	1.76	1.20–2.57	0.003

**FIGURE 2:**
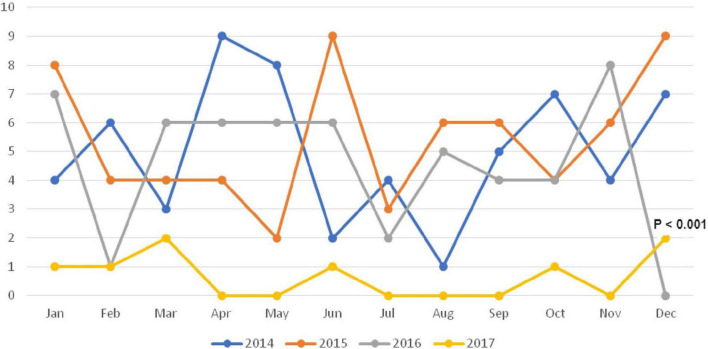
Seasonality of respiratory failure among under-five children over the study period.

The trend of incidence for respiratory failure gradually reduced over the years from 2014 to 2017 and the reduction was statistically significant in 2017 (*p* < 0.001) (Figure 2).

## Discussion

To the best of our knowledge, this was the first study that evaluated the prevalence, predicting factors, and outcomes of respiratory failure in children under 5 years of age hospitalized with the WHO classified (both the clinical and radiological) pneumonia or severe pneumonia in developing country setup. This study identified several important observations: (i) the prevalence of respiratory failure was about 5%; (ii) severe sepsis, convulsion, anemia, and severe underweight were found to be the independent risk factors for developing respiratory failure; (iii) as anticipated, mortality rate was significantly higher among those who had respiratory failure compared to those without respiratory failure; and (iv) the incidence of respiratory failure gradually reduced over the years, but the reduction was significant on 2017 than the previous years.

In this study, the prevalence of respiratory failure among the overall study population was found to be 4.58%; however, it was higher (7.22%) in patients with severe pneumonia. Although we did not have precise data on the prevalence of respiratory failure in such children, limited data revealed that the rate may range from 6 to 23% (11, 12).

The major observation of this study was the independent association of severe sepsis with respiratory failure to this study children is quite explicable. Severe sepsis is often associated with severe metabolic acidosis requiring respiratory compensation with rapid and deep breathing (26) that might exhaust oxygen stores in the lungs, as well as in the cellular level of others vital organs of the body and often culminating to global hypoxemia and potentially end up with ventilation-perfusion mismatch resulting in respiratory failure (9). Kristina E Rudd et al. had found in his analysis that there were an estimated 11 million total sepsis-related deaths, representing 19.7% of deaths worldwide in 2017. These sepsis-related deaths were attributable to lower respiratory infections, leading to respiratory failure, which was consistent with this study findings (27).

The association of convulsion with respiratory failure might be related to hypoxemia that potentially cause cellular hyperexcitability in the brain leading to convulsion (28). Higher observation of severe sepsis among the study children having respiratory failure might bolster convulsion in some children (15) in this study population who developed respiratory failure. Similar findings were observed in a study, which was conducted in a Japanese clinics and hospitals between 2012 and 2016 among the 16 million Japanese patients, who were influenza positive. Among them, 1% participants required hospitalization, where 3,361 participants developed respiratory failure and 2,603 participants had seizure disorder; the incidence was highest for influenza-positive patients aged 0–5 years and there were statistically significant decreasing trends over the years in the incidence of all causes of hospitalizations (29).

The independent association of anemia with respiratory failure is noble and interesting. Critically ill patients with hypoxemia might fail to produce interaction between oxygenation, hemoglobin level, and cardiac output that may lead to respiratory failure (30). Moreover, the oxygen-carrying capacity of red blood cell in our critically ill children with anemia might be hampered due to reduced level of hemoglobin and simultaneously, tissue hypoxemia might persist, even though pulse oximeter showed normal level (9). Thus, the patients potentially became exhausted and developed respiratory failure. One study suggesting that anemia is associated with adverse outcomes in patients receiving mechanical ventilation, including poorer survival, increased duration of mechanical ventilation, and increased reintubation rates. However, critically ill patients in respiratory failure, there is lack of the necessary replacement capacity to preserve tissue oxygenation; thus, there is development of progressive anemia among ICU patients (31).

We also observed the independent association of severe underweight with respiratory failure in this study children. A number of previous studies revealed that severely underweight children with severe illness, such as severe pneumonia, were more vulnerable to developing interstitial edema in lungs (32–34), which might lead to development of respiratory failure.

One of our important observations was that the incidence of respiratory failure gradually reduced over the years; however, the reduction was significant in 2017 compared to previous years. It is important to note that we implemented our revised guideline for the management of pneumonia or severe pneumonia, severe sepsis, and hospital-acquired infection or pneumonia in 2016 in the Dhaka Hospital of icddr,b and these interventions might had an additional impact for the significant reduction of respiratory failure in 2017 compared to the previous years.

As expected, we also observed significantly higher deaths among children with severe pneumonia having respiratory failure compared to those who did not develop respiratory failure and the observation was found to be consistent with earlier study findings (14).

Retrospective analysis of the data was the main limitation of this study, which might allow our data to have some non-standardized and missing information. Regarding the diagnostic points of respiratory failure, arterial blood gas analysis is the most accurate test to diagnose. The clinicians of resource-poor settings have performed the diagnosis of respiratory failure based on the SpO_2_/FiO_2_ ratio, where FiO_2_ was calculated adding 4% for each liter of oxygen to the FiO_2_ in room air (21%). However, we routinely audited electronic database system in the hospital from where the large data set was composed.

Large data set comprising the cohort for children having the WHO defined clinical, as well as radiological pneumonia was the strength of this study.

## Conclusion

The results of this study suggested that the prevalence of respiratory failure was 4.6% in under-five children hospitalized for pneumonia or severe pneumonia and 7.2% in severe pneumonia; however, its incidence reduced over the years. Severe sepsis, convulsion, anemia, and severe underweight have been revealed as the independent predictors of respiratory failure in children hospitalized for pneumonia or severe pneumonia. The case-fatality rate was significantly higher among children having respiratory failure than their counterpart. For better diagnosis of respiratory failure, a study with a large sample size may be undertaken that may help for the reduction of respiratory failure-related childhood deaths. Early identification of these predicting factors of respiratory failure may help our clinicians for prompt management in order to reduce potential deaths in such children, especially in resource-constrained settings.

## Data Availability Statement

The original contributions presented in the study are included in the article/supplementary material, further inquiries can be directed to the corresponding author/s.

## Ethics Statement

Ethical review and approval was not required for the study on human participants in accordance with the local legislation and institutional requirements. Written informed consent for participation was not required for this study in accordance with the national legislation and the institutional requirements.

## Author Contributions

SS and MC: conceptualization and formal analysis, and supervision. TAh and MC: investigation. TAh, AB, and MC: methodology. SS, MI, AB, and MC: data collection, analysis, and interpretation. SS, TAl, AB, LS, MS, SN, FA, YJ, GM, HS, MI, KS, SI, and TAh: writing—original draft. SS, TAh, and MC: writing—review and editing. All authors contributed to the article and approved the submitted version.

## Conflict of Interest

The authors declare that the research was conducted in the absence of any commercial or financial relationships that could be construed as a potential conflict of interest.

## Publisher’s Note

All claims expressed in this article are solely those of the authors and do not necessarily represent those of their affiliated organizations, or those of the publisher, the editors and the reviewers. Any product that may be evaluated in this article, or claim that may be made by its manufacturer, is not guaranteed or endorsed by the publisher.
